# The origin of *bmp16*, a novel *Bmp2/4 *relative, retained in teleost fish genomes

**DOI:** 10.1186/1471-2148-9-277

**Published:** 2009-12-01

**Authors:** Nathalie Feiner, Gerrit Begemann, Adina J Renz, Axel Meyer, Shigehiro Kuraku

**Affiliations:** 1 Laboratory for Zoology and Evolutionary Biology, Department of Biology, University of Konstanz, Universitätsstrasse 10, 78464 Konstanz, Germany

## Abstract

**Background:**

Whole genome sequences have allowed us to have an overview of the evolution of gene repertoires. The target of the present study, the TGFβ superfamily, contains many genes involved in vertebrate development, and provides an ideal system to explore the relationships between evolution of gene repertoires and that of developmental programs.

**Results:**

As a result of a bioinformatic survey of sequenced vertebrate genomes, we identified an uncharacterized member of the TGFβ superfamily, designated *bmp16*, which is confined to teleost fish species. Our molecular phylogenetic study revealed a high affinity of *bmp16 *to the *Bmp2/4 *subfamily. Importantly, further analyses based on the maximum-likelihood method unambiguously ruled out the possibility that this teleost-specific gene is a product of teleost-specific genome duplication. This suggests that the absence of a *bmp16 *ortholog in tetrapods is due to a secondary loss. *In situ *hybridization showed embryonic expression of the zebrafish *bmp16 *in the developing swim bladder, heart, tail bud, and ectoderm of pectoral and median fin folds in pharyngula stages, as well as gut-associated expression in 5-day embryos.

**Conclusion:**

Comparisons of expression patterns revealed (1) the redundancy of *bmp16 *expression with its homologs in presumably plesiomorphic expression domains, such as the fin fold, heart, and tail bud, which might have permitted its loss in the tetrapod lineage, and (2) the loss of craniofacial expression and gain of swim bladder expression of *bmp16 *after the gene duplication between *Bmp2*, *-4 *and *-16*. Our findings highlight the importance of documenting secondary changes of gene repertoires and expression patterns in other gene families.

## Background

In the pre-genomic era, genes were identified and sequenced in a heuristic manner to address specific biological questions. After a long period of such heuristic exploration, many genes are now comprehensively identified as parts of genomic sequences determined in large-scale projects. With several relatively well-assembled genome sequences both for teleost fishes and tetrapods, one might expect that we have discovered almost all genes commonly possessed by vertebrates (see [1]). This should be proven with care, however, especially for gene families whose members were identified and functionally characterized intensively in the very early phase of development of molecular biology. This caution is emphasized when we take into account a generalized view that diverse species possess similar gene repertoires, for well-characterized gene families, such as those containing many regulatory genes involved in animal development, namely 'toolkit genes' ([2]; also see [3]), such as *Hox *genes [4].

One of those is a group of genes containing vertebrate *bone morphogenetic proteins *(*bmp*) *2*, *bmp4*, and fly *decapentaplegic *(*dpp*) genes. These genes are members of the transforming growth factor (TGF) β superfamily, which encode proteins that are cleaved to be active extracellular signaling ligands [5,6]. The TGFβ superfamily contains other genes encoding signal proteins, such as Bmp2-15, growth differentiation factors (GDFs), TGFβ, inhibins and activins (note that Bmp1 [tolloid] is a protocollagen C-end peptidase that belongs to the Zinc-dependent metalloprotease family [7]). In vertebrates, Bmp2 and -4 are involved in various developmental processes, such as axial patterning, tissue specification and organogenesis (e.g. [8-11]; reviewed in [12]). In particular, there are several pioneering studies that highlighted the importance of this group of genes in understanding the molecular mechanisms that underlie morphological changes during evolution (e.g. [13-17]).

**Figure 1:**
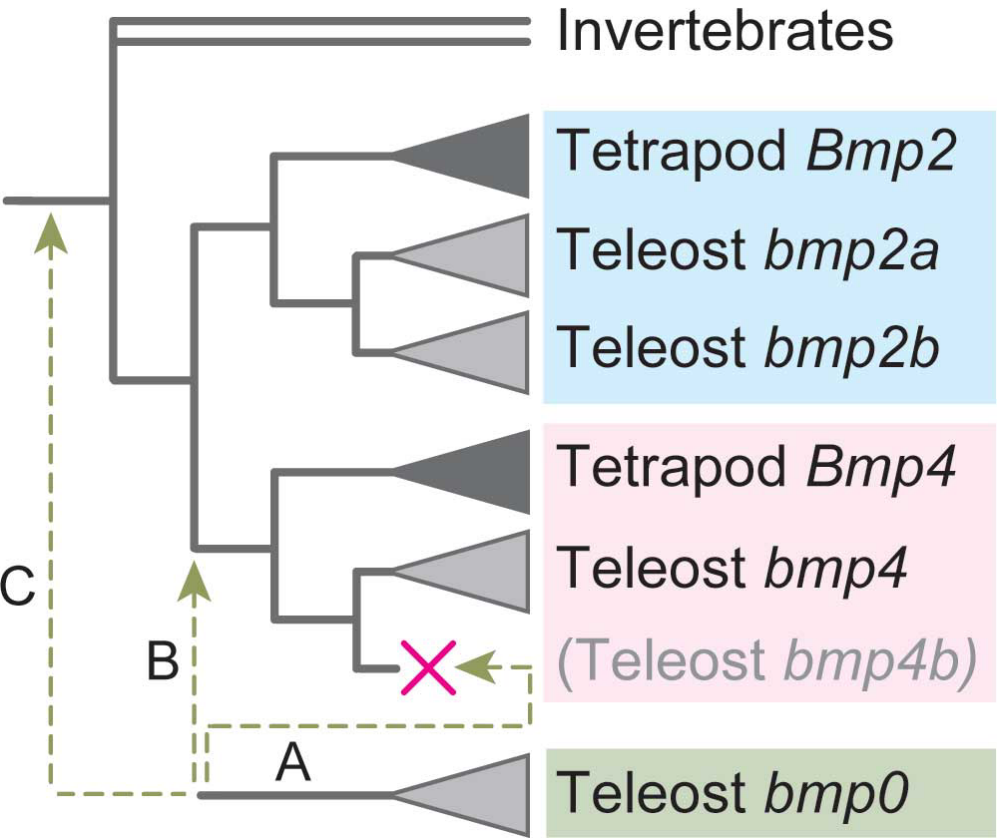
**Possible phylogenetic positions of *bmp16 *relative to those of *Bmp2 *and *Bmp4***. Because of the highly divergent nature of *bmp16 *sequences, it is not clear whether this gene was generated by the teleost-specific genome duplication (A), the two-round genome duplications early in vertebrate evolution (B), or a gene duplication event in an earlier evolutionary period (C).

In this study, we identified an uncharacterized relative of *Bmp2/4 *genes that is possessed only by teleost genomes, and designated it *bmp16*. Although additional gene repertoires unique to teleost fishes are often explained by the teleost-specific genome duplication (Figure 1A), our molecular phylogenetic analysis suggested that this gene diverged early in vertebrate evolution and is equidistant to both *Bmp2 *and *-4 *(Figure 1B). It should therefore be regarded as one of the duplicates that was generated early in vertebrate evolution, whose ortholog was lost in the lineage leading to the amniotes. Through analyzing expression patterns of the zebrafish *bmp16 *gene, we propose possible scenarios that may explain the evolution of gene repertoires and functions.

## Results

### *In silico* identification of a novel *bmp2/4* homolog in teleost genomes

**Figure 2:**
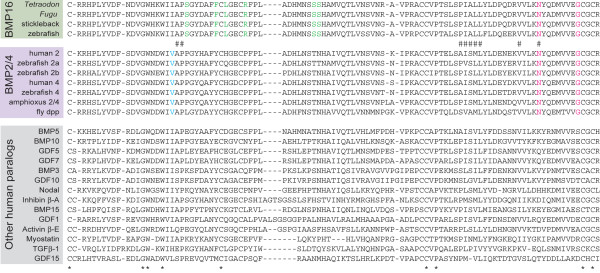
**A multiple alignment of amino acid sequences of *Bmp2/4/16 *and other members of the TGFβ superfamily**. The alignment is shown for the mature TGFβ ligand domain (SMART domain ID, SM00204). Amino acid residues conserved only among teleost bmp16 are shown in green, while those conserved in all Bmp2/4/16 sequences are shown in red. Amino acid residues conserved only in Bmp2/4 are shown in blue. Amino acid residues in the human Bmp2 that are recognized by receptors are shown with '#' [18].

A local Blastp search using the human *Bmp2 *gene as a query against downloaded available Ensembl peptide sequences for all vertebrate species produced high similarity to a group of uncharacterized peptide sequences for teleost fishes (see Table 1, for IDs of these Ensembl entries), which was later designated '*bmp16*'. The search against Ensembl peptide sequences didn't find any possible ortholog to this gene for medaka, but its genome assembly contained a significantly similar unannotated sequence on chromosome 13, at nucleotide positions 13805647-13813567 (importantly, this is neither *bmp2b *nor *-4*). For this putative medaka *bmp16 *ortholog, we also found transcript sequences, derived from embryonic cDNA libraries, in NCBI dbEST (accession numbers, [NCBI:AM318552] and [NCBI:DK098052]) that have identical sequences to this genomic region and show high similarity to *bmp16 *of other teleost fishes. Including medaka, all five model teleost fish species with whole sequenced genomes possess a single *bmp16 *gene. We detected some amino acids shared only among teleost bmp16 sequences, but overall sequences in the mature TGFβ ligand domain, which is located at the C-terminus, were highly conserved between bmp16 and other TGFβ subtypes (Figure 2).

**Table 1:** Ensembl entries for *bmp16 *genes

Species	Ensembl Gene ID	Genomic location
*Danio rerio *(zebrafish)	ENSDARG00000068180	Chromosome 18
*Gasterosteus aculeatus *(stickleback)	ENSGACG00000009621	Group I
*Oryzias latipes *(medaka)	Not annotated	Chromosome 13
*Takifugu rubripes*	ENSTRUG00000011486	Scaffold 9
*Tetraodon nigroviridis*	ENSTNIG00000007749	Chromosome 16

### Structural properties of *bmp16*

By means of RT-PCR, we amplified 5' and 3' cDNA fragments of the zebrafish *bmp16 *(see Methods for details). By assembling the two cDNA fragments, we obtained a contiguous cDNA sequence containing a small fraction of 5' UTR, full protein-coding region and a full 3' UTR, which was submitted to EMBL Nucleotide Sequence Database under the accession number [EMBL:FN400947]. In an alignment between two predicted transcript sequences currently available in Ensembl (ENSDART00000098425 and ENSDART00000098426) and our assembled *bmp16 *cDNA, we found distinct differences in nucleotide sequences, probably due to putative mis-annotation of exons in the Ensembl entries (data not shown).

We then aligned our cDNA with genomic DNA sequences retrieved from Ensembl, to find out correct exon-intron boundaries (Additional file 1: Figure S1). It turned out that the differences between our and predicted Ensembl transcripts originates partly from an incorrect assembly of genomic sequences, which is verified by our PCR amplification of the corresponding genomic region (Additional file 1: Figure S1). Our genomic sequence was also submitted to EMBL Nucleotide Sequence Database under the accession number [EMBL:FN400946].

In the deduced amino acid sequences of *bmp16*, it was found that six amino acid residues, out of nine that for human Bmp2 interact with its receptor [18], are rigidly conserved between all teleost fishes analyzed here (Figure 2).

*In silico *scan for locations of the *bmp16 *gene in the genomes of teleost fishes, except zebrafish, highlighted its linkage with *leucine-rich repeat containing *(*lrrc*) *68*, *gem-associated protein *(*gemin*) *7*, *splicing factor arginine/serine-rich *(*sfrs*) *16*, *reticulon *(*rtn*) *2 *and *relB *genes in a approximately 500-kb long genomic stretch (Additional file 2: Figure S2). In the human genome, their orthologs were found tightly clustered in a genomic stretch with a similar size on chromosome 19 (Additional file 2: Figure S2).

### Molecular phylogenetic analyses: in or out?

**Figure 3:**
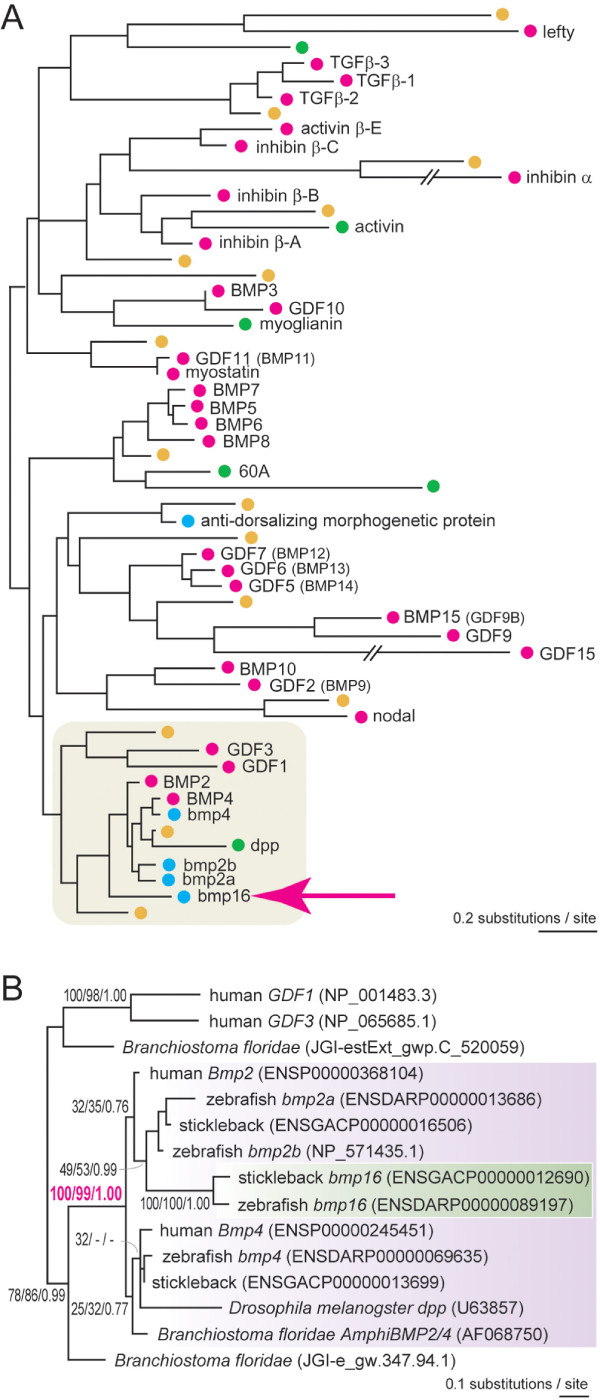
**Molecular phylogenetic tree of the TGFβ superfamily**. (A) This tree was inferred using the ML method assuming the JTT+I+Γ_4 _model (α= 0.85). Only the C-end mature ligand domain highly conserved throughout the TGFβ superfamily (62 amino acid sites) was employed in the analysis. Green, blue, red, and yellow dots indicate sequences of fly (*Drosophila melanogaster*), zebrafish, human, and amphioxus (*Branchiostoma floridae*), respectively. Note that some sequences of fly and amphioxus with many unique gaps were excluded from this analysis. All available human homologs were included. Zebrafish sequences are included only for *bmp2/4 *and *bmp16 *genes as well as anti-dorsalizing morphogenetic protein where there is no human ortholog available. (B) A molecular phylogenetic tree of *Bmp2*, *-4* and *-16*. This was estimated as the ML tree assuming the JTT+I+Γ_4 _model (α= 0.93). 82 amino acid sites were used in the analysis. Support values at nodes are shown in order, bootstrap probabilities in the ML analysis, bootstrap probabilities in the neighbor-joining analysis, and Bayesian posterior probabilities.

**Figure 4:**
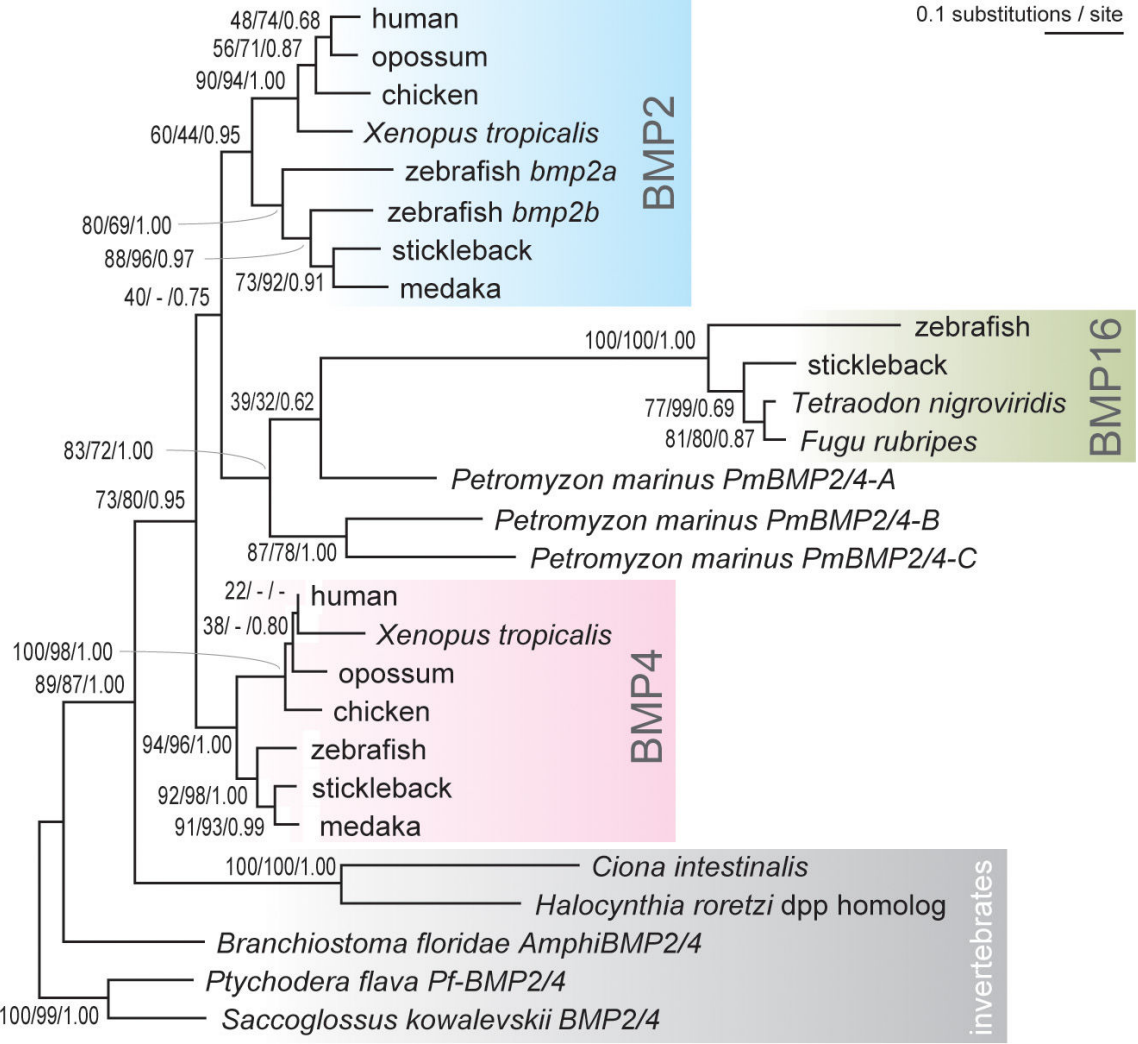
**Molecular phylogenetic tree of the *Bmp2/4/16 *subfamily**. A tree focusing on *Bmp2/4/16 *based on the ML method assuming the JTT+I+Γ_4 _model (α= 0.82). 212 amino acid sites were used in the analysis. Support values at nodes are shown in order, bootstrap probabilities in the ML analysis, bootstrap probabilities in the neighbor-joining analysis, and Bayesian posterior probabilities. See Additional file 3: Table S1 for accession IDs of the included sequences.

Our molecular phylogenetic analysis including the entire TGFβ superfamily for fly, amphioxus and human, suggested a high affinity of *bmp16 *to *Bmp2 *and *Bmp4 *(Figure 3A). This is consistent with the observation that, in the multiple alignment, some amino acids were exclusively shared between *bmp16 *and *Bmp2/4 *(Figure 2). Although this overall tree placed the *bmp16 *gene outside the *Bmp2/4 *group (Figure 3A), a restricted phylogenetic analysis based on the maximum-likelihood (ML) method with *Bmp2, -4, -16 *and their phylogenetic neighbors provided a robust support for placement of *bmp16 *inside or basal to the bilaterian *Bmp2/4 *group (Figure 3B; bootstrap probability in the ML analysis, 100; Bayesian posterior probability, 1.00). In this tree, *bmp16 *genes cluster with teleost *bmp2a/2b *genes. However, the statistical support for this grouping is low (bootstrap probability in the ML analysis, 49). We then constructed a more focused molecular phylogeny on *Bmp2*, *-4*, and *-16*, with more available sequences (Figure 4; also see additional file 3 ), including those of non-model vertebrate species, such as the sea lamprey (*Petromyzon marinus*) for which three *Bmp2/4 *homologues were already reported [19] (see also [16] for their orthologs of the Japanese lamprey *Lethenteron japonicum*). Importantly, unlike the previous tree inferences (Figure 3), this analysis allowed the employment of the amino acid alignment outside the TGFβ ligand domain that are conserved only within the *Bmp2/4 *subfamily (212 amino acid sites including the TGFβ ligand domain). The ML method supported the tree topology where *bmp16 *is clustering with one of the sea lamprey genes (namely *PmBmp2/4-A*). In this tree, *Bmp2 *is more closely related to *bmp16*/*PmBmps *than to *Bmp4*, but the statistical support is not high (bootstrap probability in the ML analysis, 40; Bayesian posterior probability, 0.75).

**Figure 5:**
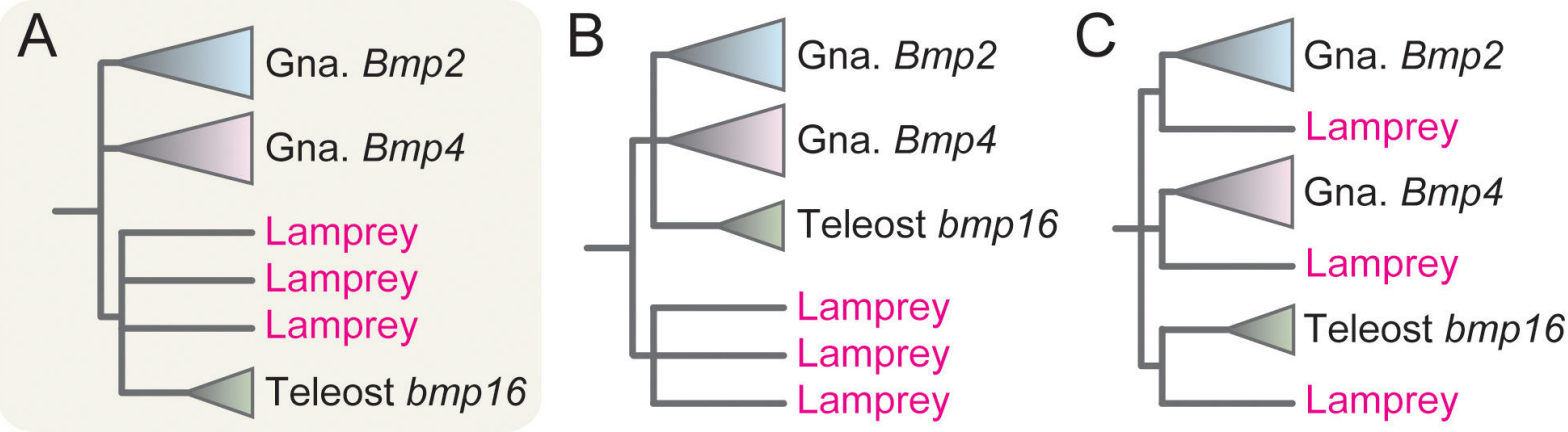
**Alternative scenarios for phylogenetic relationships between gnathostome *Bmp2/4/16 *and their lamprey homologs**. (A) The consensus tree of the eight topologies supported by our ML analysis (see Table 2 for details). Under this scenario, gene duplications that gave rise to *Bmp2*, -4, and -16 are postulated before the cyclostome-gnathostome split. This scenario also assumes additional gene duplications and losses. (B) The scenario where the cyclostome-gnathostome split occurred before the gene duplications that gave rise to three lamprey subtypes and also before gene duplications that gave rise to gnathostome *Bmp2*, -4 and -16. (C) The scenario where gene duplications that gave rise to *Bmp2*, -4, and -16 preceded the cyclostome-gnathostome split.

To assess alternative scenarios for the origin of *bmp16*, we conducted an exhaustive ML analysis with seven operational taxonomic units (OTUs) (namely, *bmp16*, *Bmp2*, *Bmp4*, *PmBmp2/4-A*, *PmBmp2/4-B*, *PmBmp2/4-C*, and outgroup; see Methods for details). Out of all 945 possible tree topologies, eight tree topologies including the ML tree have been revealed to be within 1σ of log-likelihood difference (Table 2). The consensus topology of these eight tree topologies is shown in Figure 5A. Tree topologies suggesting scenarios where all gene duplications giving rise to *Bmp2*, *-4 *and *-16 *occurred after the lamprey-gnathostome split (Figure 5B) were not supported with high statistical confidence, nor the scenario that gene duplications giving rise to *Bmp2*, -*4 *and -*16 *occurred before the lamprey-gnathostome split (Figure 5C; Table 2).

**Table 2:** Statistical supports for alternative tree topologies for relationships among *Bmp2/4/16 *genes

Rank^a^	Tree topology	Supported scenario	*Δ*log*L *± SE	*Δ*log*L*/SE	RELL BP	BPP	*P*-value	
							
							AU	SH
1	(((((PmB, PmC), (PmA, **bmp16**)), Bmp2), Bmp4), Out)	Fig. 5A	ML	0.00	0.45	0.65	0.91	1.00
2	((((((PmB, PmC), **bmp16**), PmA), Bmp2), Bmp4), Out)	Fig. 5A	1.49 ± 3.77	0.40	0.18	0.15	0.76	0.96
3	((((((PmB, PmC), **bmp16**), PmA), Bmp4), Bmp2), Out)	Fig. 5A	3.72 ± 4.68	0.80	0.04	0.02	0.43	0.86
4	(((((PmB, PmC), **bmp16**), PmA),(Bmp4, Bmp2)), Out)	Fig. 5A	3.77 ± 4.59	0.82	0.02	0.02	0.27	0.86
5	(((((PmB, PmC), (PmA, **bmp16**)), Bmp4), Bmp2), Out)	Fig. 5A	2.22 ± 2.55	0.87	0.08	0.07	0.43	0.92
6	((((((**bmp16**, PmC), PmB), PmA), Bmp2), Bmp4), Out)	Fig. 5A	5.33 ± 5.81	0.92	0.03	0.00	0.27	0.79
7	((((((**bmp16**, PmB), PmC), PmA), Bmp2), Bmp4), Out)	Fig. 5A	5.33 ± 5.81	0.92	0.03	0.00	0.27	0.79
8	((((PmB, PmC), (PmA, **bmp16**)),(Bmp2, Bmp4)), Out)	Fig. 5A	2.27 ± 2.42	0.94	0.03	0.07	0.24	0.92
								
158	(((Bmp4, (Bmp2, **bmp16**)), (PmA, (PmB, PmC))), Out)	Fig. 5B	13.81 ± 5.99	2.31	0.00	0.00	0.04	0.36
373	((((Bmp2, PmC), (**bmp16**, PmA)), (Bmp4, PmB)), Out)	Fig. 5C	30.84 ± 10.87	2.84	0.00	0.00	0.02	0.02

^a^All 945 tree topologies were sorted by *Δ*log*L*/SE. Abbreviations: Bmp2, gnathostome Bmp2; Bmp4, gnathostome Bmp4; bmp16, teleost bmp16; PmA, *P. marinus *PmBmp2/4-A; PmB, *P. marinus *PmBmp2/4-B; PmC, *P. marinus *PmBmp2/4-C; Out, outgroup; SE, standard error of *Δ*log*L*; RELL BP, Probability based on resampling of estimated log-likelihoods (RELL); BPP, Bayesian posterior probability; AU, approximate unbiased test; SH, Shimodaira-Hasegawa test. For tree topologies supporting scenarios hypothesized in Figure 5B and 5C, only those with the largest likelihood are shown, respectively.

### Gene expression analysis of *bmp16*

**Figure 6:**
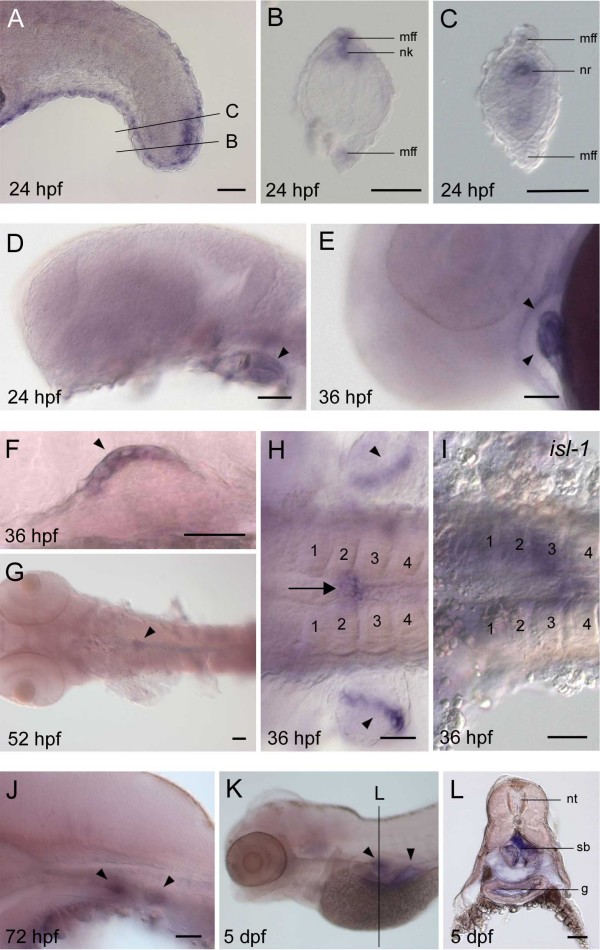
**Expression patterns of *bmp16 *in zebrafish embryos**. *In situ *hybridization at 24 (A-D), 36 (E, F, H, I), 52 (G) and 72 hpf (J) and at 5 dpf (K, L). All pictures except I (*islet-1 *[*isl-1*]) show expression of the zebrafish *bmp16*. (A) A lateral view with expression signals in a crescent-shaped region of the tail bud mesenchyme. (B, C) Transverse sections at the levels indicated in A, showing signals in the neural keel (nk) and the neural rod (nr), respectively. (D, E) Lateral views showing *bmp16 *expression (arrowheads) in the developing heart. (F) A lateral view focusing on the expression signals in the apical ectodermal fold of the pectoral fin bud (arrowhead). (G) A dorsal view of the expression domain in the developing swim bladder (arrowhead) at 52 hpf. (H) A dorsal view of the developing swim bladder (arrow) and the pectoral fin buds (arrowheads). (I) A dorsal view of the same region as in H, showing *isl-1 *expression in a more caudal region (numbers indicate somite counts). (J) A lateral view of the two distinct expression domains (arrowheads), of which the caudal one marks the developing swim bladder in a 72 hpf embryo. (K) A lateral view of a 5 dpf embryo with *bmp16 *expression signals (arrowheads). (L) A transverse section of the embryo shown in K with expression signals in the gut epithelium (g) and swim bladder (sb). The section was prepared by manually cutting the embryo using a razor blade. Other abbreviations: g, gut; mff, median fin fold; nk, neural keel; nr, neural rod; nt, neural tube; sb, swim bladder. Scale bar: 50 μm.

To characterize expression patterns of the zebrafish *bmp16 *gene, we performed *in situ *hybridization for embryos spanning from 2 hours post fertilization (hpf) to 5 days post fertilization (dpf). Expression was not detected prior to early segmentation stages and was first clearly evident at 18 hpf in the primordium of the median fin fold, where expression remained detectable until 36 hpf (data not shown). By 24 hpf, *bmp16 *is expressed in a region of the tail bud mesenchyme that dorsally and caudally flanks the midline precursor cells, i.e. the chordoneural hinge, in a crescent shaped domain (Figure 6A). Its caudalmost expression is in dorsal cells that form the neural keel, with stronger expression towards the center of the neural keel cells (Figure 6B). More anteriorly, *bmp16 *transcripts are localized to the inner extremities of the cells in the neural rod (Figure 6C). Lower levels of expression are found in cells of the emerging midline structures [20] (Figure 6C). *bmp16 *is also expressed in the developing heart, where it was detected from 24 hpf onwards and with strong expression in both chambers at 36 hpf (Figure 6D and 6E).

Between 36 and 48 hpf, *bmp16 *is expressed in the apical ectodermal fold, a specialized epithelial structure homologous to the tetrapod apical ectodermal ridge (AER), of the pectoral fins (Figure 6F and 6H). Around 36 hpf, *bmp16 *also starts to be expressed in a region of the midline endoderm at the level of the anterior extent of the pectoral fins (Figure 6H). To better characterize these cells we compared *bmp16 *expression with that of the marker for the dorsal pancreatic bud, *islet-1 *(*isl-1*; [21]), and found that *bmp16 *localizes to endodermal cells at the boundary between somites 2 and 3, while *isl-1 *is expressed slightly more caudally at the somite 3-4 boundary (Figure 6H and 6I). At 52 hpf, *bmp16 *expression continues to align with the midline (Figure 6G), and by 72 hpf is found in an additional, more anterior patch of cells that is associated with the foregut endoderm, whereas the posterior expression domain marks the developing swim bladder [22] (Figure 6J). Accordingly, at 5 dpf *bmp16 *is strongly expressed in the swim bladder as well as in the gut epithelium (Figure 6K and 6L).

## Discussion

### Timings of gene duplications between *Bmp2*, *-4* and *-16*

Since the teleost-specific genome duplication was first proposed, many genes unique to teleost genomes have been attributed to this event. This event *per se *is one of the best systems to characterize the evolutionary impact of whole genome duplications [4,23]. However, our finding of the novel *bmp16 *genes unique to teleost genomes does not align with this framework. All teleost genomes examined so far have a single ortholog (*bmp4*) to tetrapod *Bmp4*, while two *Bmp2 *orthologs (*bmp2a *and *-2b*) are found in the zebrafish. Notably, our molecular phylogenetic analyses did not place the *bmp16 *into either of *Bmp2 *or *Bmp4 *subfamilies (Figure 1B and 4). Instead, the closer relationship of *bmp16 *to three lamprey *Bmp2/4 *homologs was supported (Figure 5A). Based on statistical evaluation of likelihood values, we conclude that the origin of *bmp16 *dates back to the earliest phase of vertebrate evolution. Despite our additional attempt to characterize the timing of the gene duplication by comparing gene order in the flanking genomic regions (syntenies) between teleost *bmp16*, *-2*, and *-4*, no convincing evidence for chromosomal duplications and their timings was obtained, probably because of frequent chromosomal rearrangements in their adjacent genomic regions (data not shown). Overall, our analysis favors the scenario that all the gene duplications giving rise to *Bmp2*, *-4*, and *-16 *occurred before the cyclostome-gnathostome split. If these three subtypes are products of the so-called two-round whole genome duplications, their timing is consistent with an emerging hypothesis that the common ancestor of all extant vertebrates already experienced these events [24,25].

According to a previous study, the coverage of the available sea lamprey genome sequences should be sufficient for detecting at least one exon for any given gene [26]--consistently, fragments of the previously reported three sea lamprey *Bmp2/4 *homologs were detected. Additionally, our effort to survey publicly available sea lamprey genomic and transcriptomic sequences failed to detect additional cyclostome *Bmp2/4/16 *homologs. Therefore, after our identification of *bmp16*, both the lamprey and gnathostomes possess three *Bmp2/4/16*-related homologs. However, the most parsimonious phylogenetic scenario that postulates the smallest number of secondary gene duplications and losses (Figure 5C) was significantly rejected (Table 2). If we assume the topology of the ML tree in Figure 4 to be correct, it suggests that only *P. marinus Bmp2/4-A *is the genuine ortholog of *bmp16*, and that the gnathostome ortholog for *P. marinus Bmp2/4-B and -C *was secondarily lost in its early evolution. Accordingly, we should also assume that cyclostome orthologs for gnathostome *Bmp2 *and/or *Bmp4 *are unidentified or secondarily lost. In the light of low statistical support for the ML tree, we cannot rule out other similar hypotheses (Table 2). However, with the currently available dataset and traditional framework of molecular phylogenetics, the scenario for gene duplications and retentions of duplicates during early vertebrate evolution seems to be much more complicated than previously reported [19]. Gene sampling for other early vertebrates will be a future task. In particular, the group of hagfishes, which diverged from the future lamprey lineage just after the cyclostome-gnathostome split [27], might provide information for resolving deep divergence of *bmp16 *from others.

### *Bmp2/4/16* gene repertoires: teleost fishes and tetrapods

As well as between gnathostomes and the lamprey, our analysis has highlighted the contrast of gene repertoires between teleosts and tetrapods. In any tetrapod species for which whole genome sequences are available, *bmp16 *orthologs have not been found. The absence of this gene in the entire taxon Tetrapoda was also confirmed by our intensive survey of ESTs in NCBI. Judging from the maintenance of syntenic organization of genes surrounding the *bmp16 *gene even in species without its ortholog (Additional file 2: Figure S2), the possible loss of this gene doesn't seem to have involved deletion of a large genomic portion. The most parsimonious explanation is that the *bmp16 *ortholog was lost from the genome in a small-scale event once at the basal lineage of tetrapods. A careful scan of sarcopterygian fish genomes (namely, of lungfish and coelacanth) could narrow the time range of the gene loss.

Another interesting observation is that even though the zebrafish genome contains two *bmp2 *orthologs (namely *bmp2a *and *-2b*), other model teleost fishes categorized in Acanthomorpha seem to possess only one, namely *bmp2b *(Figure 4). Our additional survey in all available EST sequences for Acanthomorpha did not detect any more unidentified *bmp2 *relative. This suggests that *bmp2a *is confined to at least some of species in Otocephala (e.g. zebrafish, Mexican cavefish), and the *bmp2a *ortholog was secondarily lost in the lineage leading to Acanthomorpha. This line of evidence for gene losses underlines an importance of elaborate comparisons of gene repertoires even between relatively closely related animals. It is likely that more 'toolkit' genes involved in vertebrate development, which are normally considered to be present universally in all relevant genomes, are revealed to have experienced this type of evolutionary change, as shown in some previous studies (e.g. [28]).

### Roles of *bmp16* during zebrafish embryogenesis: did redundancy permit the loss?

In contrast to the divergent nature of *bmp16 *from other *Bmp2/4 *members, the deduced amino acid sequences of *bmp16 *genes seem to be relatively conserved among teleosts, especially within Acanthomorpha (Figure 2). We confirmed transcription of this uncharacterized gene in zebrafish with RT-PCR and *in situ *hybridization, and in medaka with a survey of publicly available ESTs. These data, together with the conservation of the majority of amino acid residues that are crucial for recognition by the BMP type I receptor [18], suggest that *bmp16 *functions as an active ligand in the TGFβ signaling pathway in particular biological processes.

Our analysis revealed *bmp16 *expressions in several different tissues and cell types during zebrafish embryogenesis, some of which are known to express other members of the TGFβ superfamily in tetrapods. In light of our molecular phylogenetic evidence of the loss of the *bmp16 *ortholog in the tetrapod lineage, one can ask whether its original functions might have been taken over by other members of the TGFβ superfamily in the tetrapods.

This assumption holds true for pectoral fins, because in addition to *bmp2b *and *bmp4 *[29-31], several other *bmp *genes (e.g. *bmp6, bmp7b *[32,33]) are co-expressed in the zebrafish apical ectodermal fold. In addition, expression of *bmp2a, bmp2b, bmp4 *and *bmp7 *is up-regulated in the epithelium of the developing caudal fin fold [29-32]. The AER of tetrapod limbs also expresses a number of *Bmp *genes (reviewed in [34]). Therefore, individual *Bmp *subtypes are likely to act in a redundant fashion. The loss of the *bmp16 *ortholog in the tetrapod ancestor might have been compensated in the developing specialized epithelia of median fins and paired appendages by a redundancy of *Bmp *gene function in these tissues.

Similarly, *bmp2b *and *bmp4*, as well as *bmp16*, are commonly expressed in the developing zebrafish heart [35-37]. Possible deleterious effects associated with the loss of the *bmp16 *ortholog in the tetrapod lineage could eventually have been compensated by the expression of *Bmp2 *and *Bmp4*, and the recruitment of additional homologs along with the evolution of an advanced chambering system. Accordingly, tetrapods not only express *Bmp2 *and *Bmp4 *during heart development, but also *Bmp5 *and *Bmp7 *[9,10,38,39].

Because the swim bladder and mammalian lungs share a common evolutionary origin [40], it is likely that their development is controlled by shared regulatory pathways. In the mouse, *Bmp4 *is expressed during lung development where it is initially found at high levels in the lung buds, followed by expression in the distal epithelial tips and with lower levels in the adjacent mesenchyme [41]. *Bmp4 *promotes the development of distal epithelial cell types at the expense of proximal cell types [42,43]. Accordingly, the proximal lung epithelium expresses the *Bmp4 *antagonist, *Gremlin1*, which determines proximal lung epithelial cell fate through inhibition of Bmp signaling. By regulating the proliferation, survival and morphogenetic activity of distal lung epithelial cells, Bmp signaling plays an essential role in lung morphogenesis [41,42,44-49].

In zebrafish, it has been shown that signaling through Bmp2a/2b regulates the decision between hepatic versus pancreatic fate in the endodermal midline [50,51]. Bmp signaling in adjacent lateral plate mesodermal tissues patterns the endoderm through direct contact, as disruption of coordinated cell movements between these tissues causes patterning defects in the endoderm and ceased proliferation and apoptosis of cells in the liver primordium that results from a failure of *bmp2a *expressing cells to properly contact the endoderm [51].

Comparably little is known about the roles of Bmps during swim bladder development. However, in the zebrafish, the swim bladder expresses the *protein related to Dan and Cerberus (prdc) *gene (alias: *gremlin2*; [52]), which encodes an antagonist of signaling through *bmp2a/2b *and *bmp4 *[53]. It will therefore be interesting to see whether *bmp16 *and *prdc *play comparable antagonistic roles during swim bladder development like *Bmp4*/*Gremlin1 *in the mouse lung.

Interestingly, we observed *bmp16 *expression in the tail bud between 24 and 36 hpf. Although *bmp2a *and *bmp4 *are transiently expressed in a broad domain of the caudal trunk-forming mesoderm after the end of gastrulation [30], neither gene is expressed at late stages of somitogenesis. Due to its dynamic expression in the developing neural keel, it is possible that *bmp16 *plays a role during the ongoing neurulation process in the tail, a process that probably also involves *bmp7a *[54]. Its expression in the zebrafish tail may correspond to that in the apical surface of the neural tube in tetrapods. In the mouse embryo, *Bmp2 *together with *Bmp7*, are expressed in the surface ectoderm overlying the neural folds [55]. Faure et al. showed that Smad-1, which transduces Bmp signaling, is activated in cells of the opened neural tube as well as in the ectodermal cells covering the neural folds in 10-day mouse embryos, i.e. during neurulation of the trunk region [56]. These observations support the idea that *bmp16 *in teleosts and *Bmp2 *and *-7 *in the mouse may serve similar functions during tail neurulation. Interestingly, the zebrafish *bmp16 *is expressed in partially overlapping domains with *notch1b *and *notch5 *in the tail bud mesenchyme that forms the neural rod during segmentation stages [57]. It has been shown that ectopic expression of *Bmp4 *in the *Xenopus *tail bud leads to the formation of an extra tail-like outgrowth [58]. The formation of a neural tube in this structure is Notch signaling-dependent [58]. It will therefore be of interest to define the possible genetic interactions that might exist between these signaling pathways during caudal neural keel development in the zebrafish.

### Shuffling of expression domains among *Bmp2/4/16* genes

The *bmp16 *expression patterns, scrutinized above from the developmental point of view, can also be dissected from the phylogenetic point of view to shed light on how embryonic expressions for this group of genes have been shuffled during vertebrate evolution. As for the swim bladder-associated expression of *bmp16 *(Figure 6G, H and 6J-L), our observation is the first evidence of involvement of a member of the TGFβ superfamily in its development. While neither zebrafish *bmp2a/2b *nor *bmp4 *are currently known to be expressed in the developing swim bladder [33], a focused analysis on their expression patterns in the developing endoderm at the time of swim bladder formation might be required to determine whether or not one of these genes is involved in this process. This should reveal whether other members of the bmp2/4/16 subfamily that duplicated prior to the evolution of the swim bladder were recruited for its development. Nonetheless, as described above, mouse *Bmp4 *is expressed in the developing lung, which is a homologous structure to the swim bladder [41]. So far, there is no report of lung-associated expression for *Bmp2 *or *-4 *genes in non-mammalian vertebrates. Although this might be a result of recent lineage-specific recruitment of paralogous genes in the morphogenesis of homologous structures, this endodermal expression might have already been possessed by the ancestral gene before the split of *Bmp4 *and *bmp16 *genes.

In contrast, *Bmp2 *and *-4 *expression in fin/limb buds, heart and tail bud, where zebrafish *bmp16 *is expressed (Figure 6), are found in a more taxonomically broad set of vertebrates (see [34] for limb bud expressions for various vertebrates). It is highly likely that *bmp16 *expression in those tissues was inherited from the ancestral *Bmp2/4/16 *gene, with some lineage-specific exceptions in which some expression domains are absent (e.g. absence of tail bud expression for zebrafish *bmp4*).

On the other hand, zebrafish *bmp16 *expression is absent in many of the tissues where *Bmp2 *and/or *-4 *are frequently expressed, such as in the central nervous system, otic vesicle, olfactory placode, neural crest-derived ectomesenchyme (Figure 6). Moreover, the zebrafish *bmp16 *was not expressed in pre-gastrulation stages in which *Bmp2 *and -*4 *are expressed in the ventral mesoderm to regulate dorsoventral axis specification [59]. This implies that after the gene duplication between *Bmp2*, -*4 *and -*16*, *bmp16 *expression was secondarily lost in those tissues, and was instead recruited for swim bladder development.

Even though in the molecular phylogenetic trees *bmp16 *is placed more closely to sea lamprey *Bmp2/4 *homologs than to jawed-vertebrate *Bmp2/4 *(Figure 4), a direct comparison of expression patterns between them shows that the only expression domain they have in common is the heart (between *bmp16 *and *PmBmp2/4A*) [19]. In addition to the heart, *PmBmp2/4A *is expressed in a wide variety of tissues where *Bmp2/4 *expression is usually observed in bony vertebrates, while *PmBmp2/4B *and *-C *expression is restricted to branchial arches [19]. In exploring the evolutionary history of *Bmp2/4/16 *expression patterns, this deviation in expression patterns of the sea lamprey homologs prevents us from drawing a clear-cut scenario--it would not be so surprising if early-branching lineages have accumulated a considerable number of secondary changes (see [60] for an example of cyclostome lineage-specific changes in gene order; see also [24] for a review). Information for missing early-branching lineages (e.g. chondrichthyes) might provide more clues about whether the variety of expression patterns originates from (1) frequent lineage-specific losses of expression domains from an ancestral template with a full set of domains, or (2) independent gains of similar expression domains in different lineages.

## Conclusion

Our scan in a public genome database identified a novel *Bmp2/4 *relative, *bmp16*, confined to teleost fish genomes. We suggest that this gene duplicated from *Bmp2 *and *-4 *early in vertebrate evolution, and that the absence of the tetrapod ortholog is due to the secondary loss in its basal lineage. This loss might have been permitted by the redundant expression patterns shared with other *Bmp *subtypes in fin folds, heart, and tail bud, seen in the zebrafish. At the same time, this gene was revealed to be expressed in the swim bladder, which so far has not been shown to express any other *Bmp *homolog. Our study demonstrates the power of bioinformatic, phylogenetic approaches to newly identify unexplored genes responsible for crucial biological processes. In particular, an importance of making a similar type of efforts for other gene families to document unidentified genes and secondary changes of their expression patterns is underlined.

## Methods

### Retrieval of sequences

Sequences for members of the TGFβ superfamily were retrieved from the Ensembl genome database (version 53; [61]) and NCBI Protein database, by running Blastp [62] using mammalian Bmp2 peptide sequences as queries. In parallel, peptide sequences of *Branchiostoma floridae *were also downloaded from Joint Genome institute (JGI; http://genome.jgi-psf.org/Brafl1/Brafl1.download.ftp.html), and used as a target of a Blastp search. An optimal multiple alignment of the retrieved amino acid sequences and those sequenced in this study was constructed using the alignment editor XCED, in which the MAFFT program is implemented [63].

### Molecular phylogenetic analysis

Molecular phylogenetic trees were inferred using the regions that were unambiguously aligned with no gaps. The neighbor-joining tree reconstruction [64] was processed using XCED. To calculate the support values for the molecular phylogenetic tree shown in Figures 3 and 4, we used PhyML [65] and MrBayes 3.1 [66]. Bootstrap probabilities were obtained with 100 resamplings. To investigate the phylogenetic relationship between *Bmp2/4/16 *and *P. marinus *genes (Table 2), the maximum-likelihood (ML) tree was inferred using Tree-Puzzle [67], assuming JTT+I+Γ_4 _model (shape parameter of the gamma distribution α = 0.47). In this ML analysis, we performed an exhaustive search of the ML tree in the 'user defined trees' mode of Tree-Puzzle with all 945 possible topologies consisting of seven OTUs, namely, gnathostome *Bmp2*, gnathostome *Bmp4*, teleost *bmp16*, *P. marinus PmBmp2/4-A*, *P. marinus PmBmp2/4-B*, *P. marinus PmBmp2/4-C *and outgroup. Phylogenetic relationships within individual OTUs were constrained according to generally accepted phylogenetic relationships of relevant species. Statistical tests for evaluation of alternative tree topologies were performed using CONSEL [68].

### PCR

Total RNA was extracted from a whole 52 hpf zebrafish embryo. This RNA was reverse transcribed into cDNA using SuperScript III (Invitrogen) following the instruction of 3'RACE System (Invitrogen). This cDNA was used as a template for PCR with sequence specific primers designed based on the zebrafish *bmp16 *transcript sequence found in Ensembl (ID: ENSDART00000098426). The first 3'RACE PCR was performed using the forward primer 5'-CAAGAACTGTGTTGTTTCAGCTC-3', and the product of this PCR was used as a template of the nested PCR reaction with forward primer 5'-GCTAGTCAGCTCTGTAAACGGA-3'. In amplifying the 5'-end of the *bmp16 *cDNA, we designed the reverse primer 5'-CCTCCTGGTCCAGAAAGAGT-3' based on the afore-mentioned 3'-end cDNA sequence, while the forward primer 5'-CTCCCGTTATTCCAGAGATCCA-3' was designed based on expressed sequence tags (ESTs) in NCBI dbEST (accession numbers [NCBI:CK709830], [NCBI:AW421680] and [NCBI:AW305992]) containing the 5' UTR of this gene. The assembled zebrafish *bmp16 *cDNA sequence is deposited in EMBL Nucleotide Sequence Database under the accession number [EMBL:FN400947].

Genomic DNA was extracted from 24 hpf embryos. Sequence specific primers designed based on the cDNA sequence of zebrafish *bmp16 *we obtained as above were used to amplify a 527-bp fragment containing a region where we found an incorrect assembly of genomic sequences in Ensembl (zebrafish genome assemblies, version Zv7 and Zv8) (see Additional file 1: Figure S1). This partial genomic sequence for the zebrafish *bmp16 *is deposited in EMBL Nucleotide Sequence Database under the accession number [EMBL:FN400947].

### *In situ* hybridization

The *bmp16 *riboprobes were prepared using the two cDNA fragments (5'- and 3'- fragments) amplified as above, between which we confirmed positive expression signals observed in common. In Figure 6, only results of *in situ *hybridization using the 3' probe are shown. *isl-1 *probe synthesis employed the cDNA template previously reported [69]. Whole mount *in situ *hybridization was performed as previously described [70], using the riboprobes labeled with digoxigenin-UTP (Roche Applied Science). Hybridization was detected with alkaline phosphate-conjugated anti-digoxigenin antibody followed by incubation with NBT (nitroblue tetrazolium) and BCIP (5-bromo- 4-chloro-3-indolyl phosphate). Stained embryos were examined with a Zeiss Axiophot microscope. Images were processed using Zeiss Axiovision and Adobe Photoshop software.

## Authors' contributions

SK identified *bmp16 *and performed molecular phylogenetic analyses. NF analyzed gene structure of zebrafish *bmp16 *and performed an analysis on its gene expression patterns. GB and AJR provided practical instructions about *in situ *hybridization. GB and SK wrote the first draft of the manuscript. All authors contributed to the writing of the final version of the manuscript.

## Supplementary Material

Additional file 1 **Figure S1. Sequences of teleost *bmp16 *genes**.A multiple alignment of the latest and previous assembly of the zebrafish genome sequences and sequences determined in this study. Start and stop codons are indicated with blue letters. Donor and acceptor sites at the beginning and end of introns are indicated with red letters. Exonic parts are indicated with orange background. The part in the third exon recognized as having an incorrect assembly is shown with yellow background. Genomic DNA and cDNA sequences determined in this study are deposited in EMBL under accession numbers [EMBL:FN400946] and [EMBL:FN400947], respectively. Click here for file

Additional file 2 **Figure S2. Chromosomal locations of *bmp16 *and its neighboring genes**. Gene locations are shown in contig views of the Ensembl genome browser (version 56). Zebrafish, fugu, and stickleback *bmp16 *genes are shaded in green. Orthologies of individual genes between genomes are shown with diagonal lines. Note that many genes surrounding *bmp16 *in the stickleback and fugu genomes are linked on the human chromosome 19. According to the current assembly of the zebrafish genome, *bmp16*-containing region might have experienced an additional chromosome fission/fusion event unique to its lineage, possessing another group of genes whose orthologs are located on a different genomic regions on the human chromosome 11. Click here for file

Additional file 3 **Table S1. Accession IDs for sequences in Figure 4**. Accession IDs of sequences in Figure 4 were shown with their source databases. Click here for file
