# Supplementary material for: The origin of bmp16, a novel Bmp2/4 relative, retained in teleost fish genomes
Source: BMC Evol Biol. 2009 Dec 1;9:277. doi: 10.1186/1471-2148-9-277 (PMC2801517; doi:10.1186/1471-2148-9-277)
Supplement: Additional file 1 — Figure S1. Sequences of teleost bmp16 genes.A multiple alignment of the latest and previous assembly of the zebrafish genome sequences and sequences determined in this study. Start and stop codons are indicated with blue letters. Donor and acceptor sites at the beginning and end of introns are indicated with red letters. Exonic parts are indicated with orange background. The part in the third exon recognized as having an incorrect assembly is shown with yellow background. Genomic DNA and cDNA sequences determined in this study are deposited in EMBL under accession numbers [EMBL:FN400946] and [EMBL:FN400947], respectively. [file 1471-2148-9-277-S1.PDF]

Sequence1: zebrafish bmp16-containing partial genomic sequence [EMBL: FN400946]
Sequence2: zebrafish genome assembly Zv7:chromosome 18: nt positions 43661948-43667988
Sequence3: zebrafish genome assembly Zv8:chromosome 18: nt positions 47221943-47230695
Sequence4: zebrafish bmp16 cDNA [EMBL: FN400947]

```
1 ------------------------------------------------------------------------------------------------------------------------ 1
2 ----------------------------------------------------------------------------------------tgctaaagaagaagaaaagatatataggcacc 2
3 -----------------------------------------------------------------------------------taaagtgctaaagaagaagaaaagatatataggcacc 3
4 gagaggagcgggtctgtaacgctggacaaaccatggactctgcaaaacgcccacttcacctggac------------------------------------------------------- 4
                                 exon1

1 ------------------------------------------------------------------------------------------------------------------------ 1
2 aactaaactaggacataagtttagaaatgttatgttgtctaaaccagggtttgagtctttaatctacaaaaacataattattacactggctcagtggttagcactgtcgtctcacagcag 2
3 aactaaactaggacataagtttagaaatgttatgttgtctaaaccagggtttgagtctttaatctacaaaaacataattattacactggctcagtggttagcactgtcgtctcacagcag 3
4 ------------------------------------------------------------------------------------------------------------------------ 4

1 ------------------------------------------------------------------------------------------------------------------------ 1
2 gaaggtcactggttcaagtccaattcacccatagtgcatgtcttttgactgtgggggaaaccggagaacctgaaggaaacccacgccaacacagggagaacatgccaattcacctatagt 2
3 gaaggtcactggttcaagtccaattcacccatagtgcatgtcttttgactgtgggggaaaccggagaacctgaaggaaacccacgccaacacagggagaacatgccaattcacctatagt 3
4 ------------------------------------------------------------------------------------------------------------------------ 4

1 ------------------------------------------------------------------------------------------------------------------------ 1
2 gcatgtgttttgactgtgggggaaaccagagcacctgcgggaaacccacgccaacacagggagaacatgcaaactccacacagaaatgccaactgtcccacccaggactcgaactagcga 2
3 gcatgtgttttgactgtgggggaaaccagagcacctgcgggaaacccacgccaacacagggagaacatgcaaactccacacagaaatgccaactgtcccacccaggactcgaactagcga 3
4 ------------------------------------------------------------------------------------------------------------------------ 4

1 ------------------------------------------------------------------------------------------------------------------------ 1
2 ccttcttgatgtgaggtaacagtgctaaccatgcgaactgtgccactgtgtcaccaataactttttacttgttgtcaccaaaacacactcaagaactgtgttgtttcagctcattttaac 2
3 ccttcttgatgtgaggtaacagtgctaaccatgcgaactgtgccactgtgtcaccaataactttttacttgttgtcaccaaaacacactcaagaactgtgttgtttcagctcattttaac 3
4 ------------------------------------------------------------------------------------------------------------------------ 4

                                                                                                Start
1 ------------------------------------------------------------------------------------------------------------------------ 1
2 gaaatattgtgtgctactactactactaaactactactactactaaaagtggcacctcttctttacctctctttccgcaggtccaacatgttccctgctagcctactggttctgatgat 2
3 gaaatattgtgtgctactactactactaaactactactactactaaaagtggcacctcttctttacctctctttccgcaggtccaacatgttccctgctagcctactggttctgatgat 3
4 ---------------------------------------------------------------------------------gtccaacatgttccctgctagcctactggttctgatgat 4
                                                                                                      exon2

1 ------------------------------------------------------------------------------------------------------------------------ 1
2 cctgctgctacctcaagcctcatcaggtcaccaggagggtcccagtcagactcatggaaagctggacagccttctggagcctagtttggcccataccatccagaacctcctgctaacccg 2
3 cctgctgctacctcaagcctcatcaggtcaccaggagggtcccagtcagactcatggaaagctggacagccttctggagcctagtttggcccataccatccagaacctcctgctaacccg 3
4 cctgctgctacctcaagcctcatcaggtcaccaggagggtcccagtcagactcatggaaagctggacagccttctggagcctagtttggcccataccatccagaaccttctgctaacccg 4
                                                            exon2

1 ------------------------------------------------------------------------------------------------------------------------ 1
2 cctgggcttacagtcgcaccccaaccccagtacagaggcacaagttccccagtatcttctggacttgtatcgattccacactcaacagtaccatctcatcgaggacccagaatttagtta 2
3 cctgggcttacagtcgcaccccaaccccagtacagaggcacaagttccccagtatcttctggacttgtatcgattccacactcaacagtaccatctcatcgaggacccagaatttagtta 3
4 cctgggcttacagtcgcaccccaaccccagtacaaaggcacaagttccccagtatcttctggacttgtatcgattccacactcaacagtaccatctcatcgaggacccagaatttagtta 4
                                                            exon2

1 ------------------------------------------------------------------------------------------------------------------------ 1
2 cccctcaaagcatgtgcaaggagccaacacagtccgatgctttcaccacacaggtacagcatgagcagttattgcacagtcaggagtgcccaatcctggttcctggagatctaccttcca 2
3 cccctcaaagcatgtgcaaggagccaacacagtccgatgctttcaccacacaggtacagcatgagcagttattgcacagtcaggagtgcccaatcctggttcctggagatctaccttcca 3
4 cccctcaaagcatgtgcaaggagccaacacagtccgatgctttcaccacacag------------------------------------------------------------------- 4
                             exon2

1 ------------------------------------------------------------------------------------------------------------------------ 1
2 gcaaagtccagctctgatgcaactttgatcgaacccacctgtatttaattaccaagtgctccttcgggtcctacttattttgtttagttgtgtttgatcagggttggagctgaactctgc 2
3 gcaaagtccagctctgatgcaactttgatcgaacccacctgtatttaattaccaagtgctccttcgggtcctacttattttgtttagttgtgtttgatcagggttggagctgaactctgc 3
4 ------------------------------------------------------------------------------------------------------------------------ 4

1 ------------------------------------------------------------------------------------------------------------------------ 1
2 agaaaggtagatctccaggaacaggactgggtacctaggtcatggagaacacagatttcccttggtagtcaaactaattcaggcccaatcccaattctaccacttagcccttccacttac 2
3 agaaaggtagatctccaggaacaggactgggtacctaggtcatggagaacacagatttcccttggtagtcaaactaattcaggcccaatcccaattctaccacttagcccttccacttac 3
4 ------------------------------------------------------------------------------------------------------------------------ 4

1 ------------------------------------------------------------------------------------------------------------------------ 1
2 atctacctctcgttttgcgcattcacgccaagcagtaggggcgtcccaattctcttaagcttgaaggcttagggctaaggggaacgggtgaatagccctttgaaagaagatttttacgaa 2
3 atctacctctcgttttgcgcattcacgccaagcagtaggggcgtcccaattctcttaagcttgaaggcttagggctaaggggaacgggtgaatagccctttgaaagaagatttttacgaa 3
4 ------------------------------------------------------------------------------------------------------------------------ 4

1 ------------------------------------------------------------------------------------------------------------------------ 1
2 aatagaattgggattgggccttaatttaccacaagcacagcacagagatgtgaggaggttatttaaccttctgtaggtgccaggatatgatacctgtgcattcaatattttcatatcact 2
3 aatagaattgggattgggccttaatttaccacaagcacagcacagagatgtgaggaggttatttaaccttctgtaggtgccaggatatgatacctgtgcattcaatattttcatatcact 3
4 ------------------------------------------------------------------------------------------------------------------------ 4

1 ------------------------------------------------------------------------------------------------------------------------ 1
2 ttatttcggtggtccttattgcacattatgttgaccaagttgtattgcaactacatgccacctaatttttattaaagtgtcagtatgcttaaagcagggatgggcaaacctggtcctgta 2
3 ttatttcggtggtccttattgcacattatgttgaccaagttgtattgcaactacatgccacctaatttttattaaagtgtcagtatgcttaaagcagggatgggcaaacctggtcctgta 3
4 ------------------------------------------------------------------------------------------------------------------------ 4

1 ------------------------------------------------------------------------------------------------------------------------ 1
2 gagccggtgtcctgcagagtttagcttcaaccctactaaagcacacctgcctgtagcgatattgaagacacaaattagcttgttcaggtgtgtttgattactgcagagacaccggccctt 2
3 gagccggtgtcctgcagagtttagcttcaaccctactaaagcacacctgcctgtagcgatattgaagacacaaattagcttgttcaggtgtgtttgattactgcagagacaccggccct- 3
4 ------------------------------------------------------------------------------------------------------------------------ 4

1 ------------------------------------------------------------------------------------------------------------------------ 1
2 gaggaaagagtttgcccatccagtatacttttaaatctgttggcactgccccaccaacagacattctcctgactataagaaacattgcaagtacatgttaacttaaactaatcctaacat 2
3 gaggaaagagtttgcccatccagtatacttttaaatctgttggcactgccccaccaacagacattctcctgactataagaaacattgcaagtacatgttaacttaaactaatcctaacat 3
4 ------------------------------------------------------------------------------------------------------------------------ 4
```

```
1 ------------------------------------------------------------------------------------------------------------------------ 1
2 tgcagtctaccaatactctaatgagaattatttggcatccctg-ctggaaaatccagctcaaaccagcctaggctgattgcatggttttagctggttgaccaggctggttttagaggggt 2
3 tgcagtctaccaatactctaatgagaattatttggcatccctggctggaaaatccagctcaaaccagcctaggctgattgcatggttttagctggttgaccaggctggttttagaggggt 3
4 ------------------------------------------------------------------------------------------------------------------------ 4

1 ------------------------------------------------------------------------------------------------------------------------ 1
2 tt---------------------------------------------------------------------------------------------------------------------- 2
3 ttcgagcctatatctgaccggttctccagccaattacagcgtaagataaagtgcttgtgatatgaaacgaaagggagcacaacgcagaagtacaattgattattctaatatttgcagaga 3
4 ------------------------------------------------------------------------------------------------------------------------ 4

1 ------------------------------------------------------------------------------------------------------------------------ 1
2 ------------------------------------------------------------------------------------------------------------------------ 2
3 cgaattctaccaggatcgaatggttctatataattggccgcaggaagttcactcctacgcttctggtgggagacagagacttttcatctctgaacatgaaataaaaaattgaacttattt 3
4 ------------------------------------------------------------------------------------------------------------------------ 4

1 ------------------------------------------------------------------------------------------------------------------------ 1
2 ------------------------------------------------------------------------------------------------------------------------ 2
3 gtatagaatgttcgcaagtagggtagacagaacctgactgagcttacacgtgctgtaaaattagaaatgtcctgccaaaaaaaatttacttaaagcttatgagatctactatcttcgaca 3
4 ------------------------------------------------------------------------------------------------------------------------ 4

1 ------------------------------------------------------------------------------------------------------------------------ 1
2 ------------------------------------------------------------------------------------------------------------------------ 2
3 attggaaaaactgtaagtgccccattctctaaatgcttgtcacagagaacaatgtgttgcagccaccccaaacaaaaacttaagggtgtgcggggaaaactctaaaaagtaaaaacatag 3
4 ------------------------------------------------------------------------------------------------------------------------ 4

1 ------------------------------------------------------------------------------------------------------------------------ 1
2 ------------------------------------------------------------------------------------------------------------------------ 2
3 aaactttgtgtattcagacaaaggaacaactnnnnnnnnnnnnnnnnnnnnnnnnnnnnnnnnnnnnnnnnnnnnnnnnnnnnnnnnnnnnnnnnnnnnnnnnnnnnnnnnnnnnnnnnnn 3
4 ------------------------------------------------------------------------------------------------------------------------ 4

1 ------------------------------------------------------------------------------------------------------------------------ 1
2 -----------tggccatttccaggctggtttccagccatttccagcctgatcttagctggtcaggatggaaaatgaccagctaaatccagctaaaaccaacttgaccagcctggtttaa 2
3 nnnnnnnnnnn------------------------------------------------------------------------------------------------------------- 3
4 ------------------------------------------------------------------------------------------------------------------------ 4

1 ------------------------------------------------------------------------------------------------------------------------ 1
2 gctggacaaagctggtttggnnnnnnnnnnnnnnnnnnnnnnnnnnnnnnnnnnnnnnnnnnnnnnnnnnnnnnnnnnnnnnnnnnnnnnnnnnnnnnnnnnnnnnnnnnnnnnnnnnnn 2
3 ------------------------------------------------------------------------------------------------------------------------ 3
4 ------------------------------------------------------------------------------------------------------------------------ 4

1 ------------------------------------------------------------------------------------------------------------------------ 1
2 natatatatatatatatatatatatatatatatatatatatatatatatatatatatatatatatatatatatatatatatatatatatatatatatatatatatatatatatatatata 2
3 ------------------------------------------------------------------------------------------------------------------------ 3
4 ------------------------------------------------------------------------------------------------------------------------ 4

1 ------------------------------------------------------------------------------------------------------------------------ 1
2 tatatatatatatatatatatatatatatatatatatatatatatatatatatatatatatatatatatatatatatatatatatatatatatatatatatatatatatatatatatata 2
3 -------------atatatatatatatatatatatatatatatatatatatatatatatatatatatatatatatatatatatatatatatatatatatatatatatatatatatatata 3
4 ------------------------------------------------------------------------------------------------------------------------ 4

1 ------------------------------------------------------------------------------------------------------------------------ 1
2 tatatatatatatatatatatatatatatatatatatatatatatatatatatatatatatatatatatatatatatatatatatatatatatatatatatatatatatatatatatata 2
3 tatatatatatatatatatatatatatatatatatatatatatatatatatatatatatatatatatatatatatatatatatatatatatatatatatatatatatatatatatatata 3
4 ------------------------------------------------------------------------------------------------------------------------ 4

1 ------------------------------------------------------------------------------------------------------------------------ 1
2 tatatatatatatatatatatatatatatatatatatatatatatatatatatatatatatatatatatatatatatattcttaaaccaagaagctttttcttgtaaattaaagatga 2
3 tatatatatatatatatatatatatatatatatatatatatatatatatatatatatatatatatatatatatatatattcttaaaccaagaagctttttcttgtaaattaaagatga 3
4 ------------------------------------------------------------------------------------------------------------------------ 4

1 ------------------------------------------------------------------------------------------------------------------------ 1
2 ttgtatgtttttcagaaataacgagccaaaattaagcgagtttttctttcaaacaagcaaaatgatctgccagtgggttagcaaaataatcatattctaaactaaacacaagattaggta 2
3 ttgtatgtttttcagaaataacgagccaaaattaagcgagtttttctttcaaacaagcaaaatgatctgccagtgggttagcaaaataatcatattctaaactaaacacaagattaggta 3
4 ------------------------------------------------------------------------------------------------------------------------ 4

1 ------------------------------------------------------------------------------------------------------------------------ 1
2 acactttattttgatggtctatttgagtattagtagactgtctgcttaatatgtgttaatacagacatttaactggtgtaatgtcaacttacaccagtggtgtccaaactcagtcctgga 2
3 acactttattttgatggtctatttgagtattagtagactgtctgcttaatatgtgttaatacagacatttaactggtgtaatgtcaacttacaccagtggtgtccaaactcagtcctgga 3
4 ------------------------------------------------------------------------------------------------------------------------ 4

1 ------------------------------------------------------------------------------------------------------------------------ 1
2 gggccggtgtcctggagagtttagctccaaccctaatcgaacacacatgaaccagataatcaagctctttgtagatatactagaaacttcctgcaggtgttttgaagcaagttagagcta 2
3 gggccggtgtcctggagagtttagctccaaccctaatcgaacacacatgaaccagataatcaagctctttgtagatatactagaaacttcctgcaggtgttttgaagcaagttagagcta 3
4 ------------------------------------------------------------------------------------------------------------------------ 4

1 ------------------------------------------------------------------------------------------------------------------------ 1
2 aattcagccggacaccagccctccaggactgagtttggacatgcctgacttgcactaaccctaactccaacctaagagtctactgataatctaatgtagggctgctcaaccctgttcttg 2
3 aattcagccggacaccagccctccaggactgagtttggacatgcctgacttgcactaaccctaactccaacctaagagtctactgataatctaatgtagggctgctcaaccctgttcttg 3
4 ------------------------------------------------------------------------------------------------------------------------ 4

1 ------------------------------------------------------------------------------------------------------------------------ 1
2 gagatctaccttcctgcaaagttcaggtccaaccctggtcaacacacctgaaccaattaattaggacttgaacagcacttgataaatacaggcaggtgtgtttgataagggttgcaactg 2
3 gagatctaccttcctgcaaagttcaggtccaaccctggtcaacacacctgaaccaattaattaggacttgaacagcacttgataaatacaggcaggtgtgtttgataagggttgcaactg 3
4 ------------------------------------------------------------------------------------------------------------------------ 4
```

```
1 ------------------------------------------------------------------------------------------------------------------------ 1
2 aaatctgcaggaaggtcgatctccaggaacaggattgagcacactggatctaatgagaattagttggcaattagatgcaatgtaacttaaattcaacaaacggaccatcaaaataaagag 2
3 aaatctgcaggaaggtcgatctccaggaacaggattgagcacactggatctaatgagaattagttggcaattagatgcaatgtaacttaaattcaacaaacggaccatcaaaataaagag 3
4 ------------------------------------------------------------------------------------------------------------------------ 4

1 ------------------------------------------------------------------------------------------------------------------------ 1
2 agacacaagattatttgtttgttttaagaaaaatctcacttaacttttcctgaaaacaagacaatattttaattgtctagaaaatgcttcttgatttaagaattttagattagatgta 2
3 agacacaagattatttgtttgttttaagaaaaatctcacttaacttttcctgaaaacaagacaatattttaattgtctagaaaatgcttcttgatttaagaattttagattagatgta 3
4 ------------------------------------------------------------------------------------------------------------------------ 4

1 ------------------------------------------------------------------------------------------------------------------------ 1
2 catttacattgagtcatttagcagacgcttttatccaaagcgacttacaaatgaggacaaggaagcaatttacacaactataagagctttttatttttgtagaagagttagcggtaga 2
3 catttacattgagtcatttagcagacgcttttatccaaagcgacttacaaatgaggacaaggaagcaatttacacaactataagagctttttatttttgtagaagagttagcggtaga 3
4 ------------------------------------------------------------------------------------------------------------------------ 4

1 ------------------------------------------------------------------------------------------------------------------------ 1
2 accagagaggcaattgcagattaggaagtgaagtggagactaaatagttgatttttagtcgtttcttgaagacagcgagtgactctgccgttctgatgcagttcgggagttcattccac 2
3 accagagaggcaattgcagattaggaagtgaagtggagactaaatagttgatttttagtcgtttcttgaagacagcgagtgactctgccgttctgatgcagttcgggagttcattccac 3
4 ------------------------------------------------------------------------------------------------------------------------ 4

1 ------------------------------------------------------------------------------------------------------------------------ 1
2 caactgggcagattgaacgcgagcgttcgggaaagtgatttcttccctctttgagatggaaccttgaggcgaggttcattcacagaatgtaagttactggagggcgcatacatctgcaga 2
3 caactgggcagattgaacgcgagcgttcgggaaagtgatttcttccctctttgagatggaaccttgaggcgaggttcattcacagaatgtaagttactggagggcgcatacatctgcaga 3
4 ------------------------------------------------------------------------------------------------------------------------ 4

1 ------------------------------------------------------------------------------------------------------------------------ 1
2 agtgagagcagataagagaaaaccagaagtcactttgtaagcaaacatcgaatttgatgcgagcagcaactggcagccagtgcaaacggatgagcagcggagtgacatgtgctctttcag 2
3 agtgagagcagataagagaaaaccagaagtcactttgtaagcaaacatcgaatttgatgcgagcagcaactggcagccagtgcaaacggatgagcagcggagtgacatgtgctctttcag 3
4 ------------------------------------------------------------------------------------------------------------------------ 4

1 ------------------------------------------------------------------------------------------------------------------------ 1
2 cttcattaaagaccactcgtgctgctgcgttctgaagcatctgaagaggtttgatagagttagctgggagcccggctagtagagagttgcaatagtccagtctggagagaacaagagctt 2
3 cttcattaaagaccactcgtgctgctgcgttctgaagcatctgaagaggtttgatagagttagctgggagcccggctagtagagagttgcaatagtccagtctggagagaacaagagctt 3
4 ------------------------------------------------------------------------------------------------------------------------ 4

1 ------------------------------------------------------------------------------------------------------------------------ 1
2 gaacaaggagttgagctgcatgttcagataggaagggtcggacctttctaacttagactagaaaaaaggcaaaagctctaattataaaacacatttttttgcagtgaacatgatgaaatg 2
3 gaacaaggagttgagctgcatgttcagataggaagggtcggacctttctaacttagactagaaaaaaggcaaaagctctaattataaaacacatttttttgcagtgaacatgatgaaatg 3
4 ------------------------------------------------------------------------------------------------------------------------ 4

1 ------------------------------------------------------------------------------------------------------------------------ 1
2 caagcattttacaacttaagagctttctttttttccattcagattctccgactccgagtcttccagaagagcagaagaccacagtggacggcatccacataggcttcaacctgtcctccat 2
3 caagcattttacaacttaagagctttctttttttccattcagattctccgactccgagtcttccagaagagcagaagaccacagtggacggcatccacataggcttcaacctgtcctccat 3
4 -----------------------------------------attctccgactccgagtcttccagaagagcagaagaccacagtggacggcatccacataggcttcaacctgtcctccat 4
                                              exon3

1 ------------------------------------------------------------------------------------------------------------------------ 1
2 tccttcagaagaaagtgtggtgtccgcagagttacgtctactccatgaagggtcaagtggaggttctcacgtggccagtctttacctttccaaccatgacccttcatcaaaacccattct 2
3 tccttcagaagaaagtgtggtgtccgcagagttacgtctactccatgaagggtcaagtggaggttctcacgtggccagtctttacctttccaaccatgacccttcatcaaaacccattct 3
4 tccttcagaagaaagtgtggtgtccgcagagttacgtctactccatgaagggtcaagtggaggttctcacgtggccagtctttacctttccaaccatgacccttcatcaaaacccattct 4
                                              exon3

1 ------------------------------------------------------------------------------------------------------------------------ 1
2 gctccattcacgtcaactgaccagagacaggaagagtgcccaactctgggaaacctttcccctggatagagaagtgtttcagaatttatccaagacgtcaggaagcctgtcttttatcct 2
3 gctccattcacgtcaactgaccagagacaggaagagtgcccaactctgggaaacctttcccctggatagagaagtgtttcagaatttatccaagacgtcaggaagcctgtcttttatcct 3
4 gctccattcacgtcaactgaccagagacaggaagagtgcccaactctgggaaacctttcccctggatagagaagtgtttcagaatttatccaagacgtcaggaagcctgtcttttatcct 4
                                              exon3

1 ------------------------------------------------------------------------------------------------------------------------ 1
2 ggatgttattcctgataataacagcagcagtactcttcctaaagacagacatttgcgagtacgcagatccacattgcaggacgggcctacttgggaaaggcaaaggcctttattagtgac 2
3 ggatgttattcctgataataacagcagcagtactcttcctaaagacagacatttgcgagtacgcagatccacattgcaggacgggcctacttgggaaaggcaaaggcctttattagtgac 3
4 ggatgttattcctgatagtaacagcagcagtactcttcctaaagacagacatttgcgagtacgcagatccacattgcaggacgggcctacttgggaaaggcaaaggcctttattagtgac 4
                                              exon3

1 ------------------------------------------------------------------------------------------------------------------------ 1
2 ctacagtcacgatggccgcagcgagccttttgtgaatctcaataagcgtaaaagcagccgaagcaggagcagatggaccaggtttaagggtgataggggtcaggctctgactggaatga 2
3 ctacagtcacgatggccgcagcgagccttttgtgaatctcaataagcgtaaaagcagccgaagcaggagcagatggaccaggtttaagggtgataggggtcaggctctgactggaatga 3
4 ctacagtcacgatggccgcagtgagccttttgtgaatctcaataagcgtaaaagcagccgaagcaggagcagatggaccaggtttaagggtgataggggtcaggctctgactggaatga 4
                                              exon3

1 ------------------------------------------------------------------------------------------------------------------------ 1
2 aaggcgagtgaaacgaaacaacaggagggccgcaaaactcaaaagactctcccgcgctcgttgtcgtcggcacccgctgtacgtggacttcaaagatgtcggctggaataaatggatcat 2
3 aaggcgagtgaaacgaaacaacaggagggccgcaaaactcaaaagactctcccgcgctcgttgtcgtcggcacccgctgtacgtggacttcaaagatgtcggctggaataaatggatcat 3
4 aaggcgagtgaaacgaaacaacaggagggccgcaaaactcaaaagactctcccgcgctcgttgtcgtcggcacccgctgtacgtggacttcaaagatgtcggctggaataaatggatcat 4
                                              exon3

1 -------------------------------------------cgcttcccactggctgaccatat------------------------------------------------------ 1
2 agctccatctggttacgatgctttcttttgcctcggagagtgccgcttcccactggctgaccatatgaattcatcaagccatgccatggtgcagacgctagtcagctctgtaaacggagc 2
3 agctccatctggttacgatgctttcttttgcctcggagagtgccgcttcccactggctgaccatatg----------------------------------------------------- 3
4 agctccatctggttacgatgctttcttttgcctcggagagtgccgcttcccactggctgaccatat------------------------------------------------------ 4
                             exon3

1 ------------------------------------------------------------------------------------------------------------------------ 1
2 cgtgccgcgaccatgctgcgtccctacagctctcagcccaatcgctctactctttctggaccaggaggaacgtgtggtgttaaaaactaccaagacannnnnnnnnnnnnnnnnnnnnnn 2
3 --------------------------------------------------------------------------------------------------nnnnnnnnnnnnnnnnnnnnnn 3
4 ------------------------------------------------------------------------------------------------------------------------ 4

1 ------------------------------------------------------------------------------gaattcatcaagccatgccatggtgcagacgctggtcagctc 1
2 nnnnnnnnnnnnnnnnnnnnnnnnnnnnnnnnnnnnnnnnnnnnnnnnnnnnnnnnnnnnnnnnnnnnnnnnnnnnnngaattcatcaagccatgccatggtgcagacgctagtcagctc 2
3 nnnnnnnnnnnnnnnnnnnnnnnnnnnnnnnnnnnnnnnnnnnnnnnnnnnnnnnnnnnnnnnnnnnnnnnnnnnnnngaattcatcaagccatgccatggtgcagacgctagtcagctc 3
4 ------------------------------------------------------------------------------gaattcatcaagccatgccatggtgcagacgctagtcagctc 4
                                                                                        exon3
```

```
1 tgtaaacggagccgtgccgcgaccatgctgcgtccctacagctctcagcccaatcgctctactctttctggaccaggaggaacgtgtggtgttgaaaaactaccaagacatggtggtgga 1
2 tgtaaacggagccgtgccgcgaccatgctgcgtccctacagctctcagcccaatcgctctactctttctggaccaggaggaacgtgtggtgttgaaaaactaccaagacatggtggtgga 2
3 tgtaaacggagccgtgccgcgaccatgctgcgtccctacagctctcagcccaatcgctctactctttctggaccaggaggaacgtgtggtgttgaaaaactaccaagacatggtggtgga 3
4 tgtaaacggagccgtgccgcgaccatgctgcgtccctacagctctcagcccaatcgctctactctttctggaccaggaggaacgtgtggtgttgaaaaactaccaagacatggtggtgga 4
```

exon3

```
                    Stop
1 gggatgtggctgtcgatagccagatacttgtggaataaaccagaaatggtggacctgaattaggaagaaagaaactgaaagagatgaagaaagcgaggtaaatggacacgagaaaygtaa 1
2 gggatgtggctgtcgatagccagatacttgtggaataaaccagaaatggtggacctgaattaggaagaaagaaactgaaagagatgaagaaagcgaggtaaatggacacgagaaatgtaa 2
3 gggatgtggctgtcgatagccagatacttgtggaataaaccagaaatggtggacctgaattaggaagaaagaaactgaaagagatgaagaaagcgaggtaaatggacacgagaaatgtaa 3
4 gggatgtggctgtcgatagccagatacttgtggaataaaccagaaatggtggacctgaattaggaagaaagaaactgaaagagatgaagaaagcgaggtaaatggacacgagaaacgtaa 4
```

exon3

```
1 tcctccggaacttagctttgacttcttgtgtccaatgaaacattgtatttgtgtgaactttgacccttagaacagtgattcccaacrctactcctggaggcacaccaacagtacatattt 1
2 tcctccggaacttagctttgacttcttgtgtccaatgaagcattgtatttgtgtgaactttgacccttagaacagtgattcccaacgctactcctggaggcacaccaacagtacatattt 2
3 tcctccggaacttagctttgacttcttgtgtccaatgaagcattgtatttgtgtgaactttgacccttagaacagtgattcccaacgctactcctggaggcacaccaacagtacatattt 3
4 tcctccggaacttagctttgacttcttgtgtccaatgaagcattgtatttgtgtgaactttgacccttagaacagtgattcccaacgctactcctggaggcacaccaacagtacatattt 4
```

exon3

```
1 gggatgtctcccttatctataaccttcaagttttggagtttctacaaatgttctgatgagttaattcagatgtgtttgattaggaagaggttaacatgttgg------------------ 1
2 gggatgtctcccttatctataaccttcaagttttggagtttctacaaatgttctgatgagttaattcaggtgtgtttgattaggaagaggttaacatgttggagtcccttcgggaacagg 2
3 gggatgtctcccttatctataaccttcaagttttggagtttctacaaatgttctgatgagttaattcaggtgtgtttgattaggaagaggttaacatgttggagtcccttcgggaacagg 3
4 gggatgtctcccttatctataaccttcaagttttggagtttctacaaatgttctgatgagttgattcaggtgtgtttgattaggaagaggttaacatgttggagtcccttctggaacagg 4
```

exon3

```
1 ------------------------------------------------------------------------------------------------------------------------ 1
2 gttgggaaacactgccttagaagcactcaagacgtgttgtgtctgtcttattagtacactcctgctttttctgtcttagctgtgcaatctttcctttgttgtgtaagtccttctctcgtc 2
3 gttgggaaacactgccttagaagcactcaagacgtgttgtgtctgtcttattagtacactcctgctttttctgtcttagctgtgcaatctttcctttgttgtgtaagtccttctctcgtc 3
4 gttgggaaacactgccttagaagcactcaagacgtgttgtgtctgtcttattagtacactcctgctttttctgtcttagctgtgcaatctttcctttgttgtgtaagtccttctctcgtc 4
```

exon3

```
1 ------------------------------------------------------------------------------------------------------------------------ 1
2 atcgacaactcaaagggaccaagaattgactcttgctgagcgtttctgctgccggacagacttaaaccggaggaagaagagtgaactcttctgtgaaatctctggtcgcaaatgaatctt 2
3 atcgacaactcaaagggaccaagaattgactcttgctgagcgtttctgctgccggacagacttaaaccggaggaagaagagtgaactcttctgtgaaatctctggtcgcaaatgaatctt 3
4 atcgacaactcaaagggaccaagaattgactcttgctgagcgtttctgctgccggacagacttaaaccggaggaagaagagtgaactcttctgtgaaatctctggtcgcaaatgaatctt 4
```

```
1 ---------------------------------------- 1
2 cagagtaatgcactgtgac--------------------- 2
3 cagagtaatgcactgtgac--------------------- 3
4 cagagtaatgcactgtgacagagacgataactgattgcag 4
```

exon3
