# Supplementary material for: The origin of bmp16, a novel Bmp2/4 relative, retained in teleost fish genomes
Source: BMC Evol Biol. 2009 Dec 1;9:277. doi: 10.1186/1471-2148-9-277 (PMC2801517; doi:10.1186/1471-2148-9-277)
Supplement: Additional file 2 — Figure S2. Chromosomal locations of bmp16 and its neighboring genes. Gene locations are shown in contig views of the Ensembl genome browser (version 56). Zebrafish, fugu, and stickleback bmp16 genes are shaded in green. Orthologies of individual genes between genomes are shown with diagonal lines. Note that many genes surrounding bmp16 in the stickleback and fugu genomes are linked on the human chromosome 19. According to the current assembly of the zebrafish genome, bmp16-containing region might have experienced an additional chromosome fission/fusion event unique to its lineage, possessing another group of genes whose orthologs are located on a different genomic regions on the human chromosome 11. [file 1471-2148-9-277-S2.PDF]

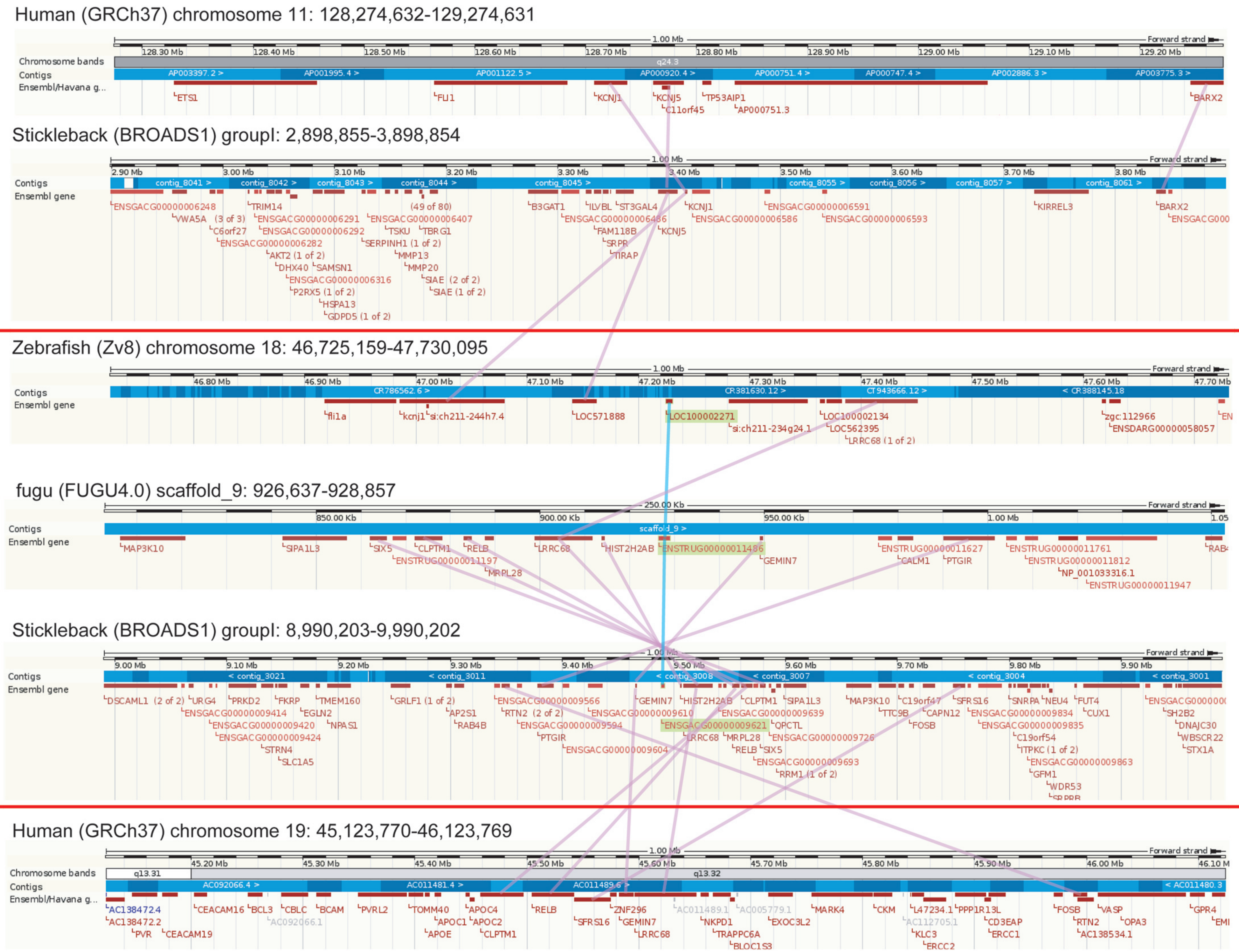

Human (GRCh37) chromosome 11: 128,274,632-129,274,631
Chromosome bands
Contigs
Ensembl/Havana g...
ETS1
FLI1
KCNJ1
KCNJ5
C11orf45
TP53AIP1
AP000751.3
BARX2
Stickleback (BROADS1) groupI: 2,898,855-3,898,854
Contigs
Ensembl gene
Zebrafish (Zv8) chromosome 18: 46,725,159-47,730,095
Contigs
Ensembl gene
fugu (FUGU4.0) scaffold_9: 926,637-928,857
Contigs
Ensembl gene
Stickleback (BROADS1) groupI: 8,990,203-9,990,202
Contigs
Ensembl gene
Human (GRCh37) chromosome 19: 45,123,770-46,123,769
Chromosome bands
Contigs
Ensembl/Havana g...
Forward strand
