# Supplementary material for: The origin of bmp16, a novel Bmp2/4 relative, retained in teleost fish genomes
Source: BMC Evol Biol. 2009 Dec 1;9:277. doi: 10.1186/1471-2148-9-277 (PMC2801517; doi:10.1186/1471-2148-9-277)
Supplement: Additional file 3 — Table S1. Accession IDs for sequences in Figure 4. Accession IDs of sequences in Figure 4 were shown with their source databases. [file 1471-2148-9-277-S3.PDF]

Table S1. Accession IDs for sequences in the Figure 4.

Species	Gene name	Database	Accession ID
human	*Bmp2*	Ensembl	ENSP00000368104
opossum	*Bmp2*	Ensembl	ENSMODP00000004813
chicken	*Bmp2*	NCBI	AY237249
Xenopus tropicalis	*Bmp2*	Ensembl	ENSXETP00000012124
zebrafish	*bmp2a*	Ensembl	ENSDARP00000013686
zebrafish	*bmp2b*	Ensembl	ENSDARP00000091255
stickleback	*bmp2b*	Ensembl	ENSGACP00000016506
medaka	*bmp2b*	Ensembl	ENSORLP00000012259
human	*Bmp4*	Ensembl	ENSP00000245451
Xenopus tropicalis	*Bmp4*	Ensembl	ENSXETP00000018837
opossum	*Bmp4*	Ensembl	ENSMODP00000016205
chicken	*Bmp4*	NCBI	X75915
zebrafish	*Bmp4*	Ensembl	ENSDARP00000069635
stickleback	*Bmp4*	Ensembl	ENSGACP00000013699
medaka	*Bmp4*	Ensembl	ENSORLP00000016673
zebrafish	*bmp16*	Ensembl	ENSDARP00000089197
stickleback	*bmp16*	Ensembl	ENSGACP00000012690
Tetraodon nigroviridis	*bmp16*	Ensembl	ENSTNIP00000010567
Takifugu rubripes	*bmp16*	Ensembl	ENSTRUP00000029004
Petromyzon marinus	*PmBmp2/4-A*	NCBI	AY602220
Petromyzon marinus	*PmBmp2/4-B*	NCBI	AY602221
Petromyzon marinus	*PmBmp2/4-C*	NCBI	AY602222
Ciona intestinalis		Ensembl	ENSCINP00000003062
Halocynthia roretzi	*dpp homolog*	NCBI	D85464
Branchiostoma floridae	*AmphiBmp2/4*	NCBI	AF068750
Ptychodera flava	*Pf-Bmp2/4*	NCBI	AB028219
Saccoglossus kowakevskii	*Bmp2/4*	NCBI	DQ431030
